# Direct Observation of Structural Phase Transformations during Phosphorene Formation on Cu(111)

**DOI:** 10.1021/acsnano.4c11802

**Published:** 2025-01-22

**Authors:** Jiří David, František Jeřábek, Pavel Procházka, Miroslav Černý, Cristian V. Ciobanu, Stanislav Průša, Tomáš Šikola, Suneel Kodambaka, Miroslav Kolíbal

**Affiliations:** † Institute of Physical Engineering, Brno University of Technology, Technická 2, 616 69 Brno, Czech Republic; ‡ CEITEC BUT, Brno University of Technology, Purkyňova 123, 612 00 Brno, Czech Republic; § Department of Mechanical Engineering, Materials Science Program, Colorado School of Mines, Golden, Colorado 80401, United States; ∥ Department of Materials Science and Engineering, Virginia Polytechnic Institute and State University, Blacksburg, Virginia 24061, United States

**Keywords:** 2D materials, phosphorene, copper phosphide, phase transformation, growth mode

## Abstract

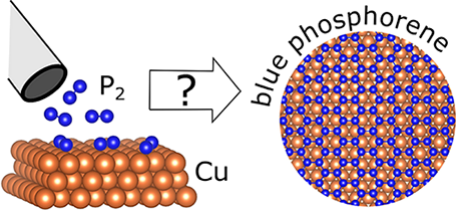

Blue phosphorene, a two-dimensional, hexagonal-structured, semiconducting phosphorus, has gained attention as it is considered easier to synthesize on metal surfaces than its allotrope, black phosphorene. Recent studies report different structures of phosphorene, for example, on Cu(111), but the underlying mechanisms of their formation are not known. Here, using a combination of in situ ultrahigh vacuum low-energy electron microscopy and in vacuo scanning tunneling microscopy, we determine the time evolution of the surface structure and morphology during the deposition of phosphorus on single-crystalline Cu(111). We find that during the early stages of deposition phosphorus intermixes with Cu, resulting in copper phosphide structures. With the increasing surface concentration of phosphorus, the phosphide phase disappears, and a blue phosphorene layer forms, followed by the self-assembly of highly ordered phosphorus clusters that eventually grow into multilayer islands. We attribute the unexpected transformation of stable phosphide to a phosphorene layer to the presence of a large concentration of P_2_ dimers on the surface. Our results constitute direct evidence for a growth mode leading to a flat phosphorene layer via an intermediary phase, which could underpin the growth of other 2D materials on strongly interacting substrates.

The realm of two-dimensional (2D) materials is a very attractive field of research due to the exceptional and often unusual properties of these materials resulting from quantum confinement in two dimensions. Large-scale fabrication of even simple elemental 2D materials can be a significant challenge, as their structure and, hence, electronic properties may differ depending on the preparation technique. A typical example is phosphorene, a monolayer of phosphorus atoms whose allotropes include black and blue phosphorene (BlackP and BlueP). The BlackP monolayer is buckled on the atomic scale and crystallizes in an orthorhombic lattice whose structural asymmetry causes an anisotropic optical response, anisotropic charge carrier mobility, etc.^1,2^ These appealing properties have driven significant efforts for the scalable fabrication of BlackP,^3,4^ which is commonly obtained by mechanical exfoliation from a bulk crystal. Blue phosphorene (BlueP) is also atomically buckled, resembles graphene due to its honeycomb structure, and exhibits properties different from BlackP.^5^ It has been predicted theoretically^6,7^ and with limited success experimentally observed^8−13^ that phosphorus evaporated onto a metal substrate can form BlueP.

The idea of the growth of 2D layers such as BlackP and BlueP on metallic single-crystals is attractive because metal substrates can be prepared on a large scale,^14^ which could lead to large-area 2D phosphorene layers as well. A potential disadvantage of metallic substrates, however, is that the interaction of the electronic energy bands of the 2D material with the substrate may negatively impact the properties, prevent exfoliation,^15^ or further processing of the 2D layers. To date, there are no reports of BlackP growth on metals. Density functional theory (DFT) simulations have predicted that BlueP layers are stable on weakly interacting substrates and that clusters of phosphorus^7,16^ or metal phosphides,^17^ rather than BlueP, are stable on strongly interacting metals. Experimentally, the growth of BlueP layers seems challenging even on Au(111), which is commonly considered to be a weakly interacting substrate. Initial reports^8,9^ of BlueP formation on Au(111) were encouraging, however, later studies^18,19^ argued that an Au–P alloy, instead of BlueP, was formed on Au(111). Interestingly, intercalation of Si and Te at the Au–P/Au(111) interfaces appears to yield BlueP.^10,11^ Successful growths of BlueP have been reported on oxidized Cu(111)^20^ and also on bare Cu(111): Kaddar et al.^12^ have claimed the formation of a buckled BlueP with a lattice constant of 3.4 Å that exhibits linear dispersion at specific points of the Brillouin zone, as expected for BlueP. On the contrary, Song et al.^13^ have demonstrated the growth of an ultraflat and chiral BlueP with a lattice constant of 4.1 Å on the same substrate. Both studies conclude that further deposition of phosphorus does not lead to multilayer growth but results in a self-limited hexagonal array of 3D phosphorus islands on top of the BlueP. While the mechanisms leading to the differences in the structure (buckled vs flat) of BlueP on Cu(111) have not been identified in these two reports, a recent theoretical study^21^ has suggested that the growth structure may depend on the chemical potential of the depositing phosphorus. Additionally, it is puzzling that BlueP layers are formed directly on Cu(111), a relatively strongly interacting metal compared to Au(111).

Here, we provide *direct* evidence of a hexagonal-structured phosphorus monolayer *and* multilayers on Cu(111), and we identify the associated growth mechanism. Using in situ low-energy electron microscopy and diffraction (LEEM and LEED) during the deposition of phosphorus on Cu(111) and scanning tunneling microscopy (STM), we follow the surface structural and morphological evolution as a function of time. At low phosphorus coverages, copper phosphides form on the surface. At later times, surprisingly, we observe an abrupt transformation of the phosphide to a blue phosphorene overlayer. Further deposition leads to the formation of a hexagonal array of phosphorus clusters on top of this phosphorene layer, followed by the coalescence of these clusters into 3D high-aspect-ratio mounds and 2D triangular islands. Our results are surprising because (1) the phosphide phase, expected to be stable on Cu(111), transforms into phosphorene and (2) multilayer islands are formed (not reported yet). We suggest (and provide justification below) that the destabilization of the phosphide occurs in the presence of a large concentration of P_2_ dimers on the surface.

## Results

In all our experiments, phosphorus is generated predominantly as P_2_ molecules by decomposing GaP in a Knudsen cell operated at high temperatures (∼1080 K) (documented in Supporting Information, Figure S1). The rate of deposition is estimated (see Methods for details) at one monolayer (ML) of P atoms per 26 min on a Cu(111) substrate held at temperature *T* = 450 K (0.038 ML/min). With increasing substrate temperature *T* up to ∼660 K, the P deposition rates decrease, presumably due to the increased desorption rate of P (and vice versa). However, the structural quality of the deposits as indicated by the LEED spot sharpness and intensity improves with increasing *T*; at *T* > 660 K, LEED shows only Cu(111)-related spots, and the LEEM images reveal surface steps characteristic of bare Cu. Low energy ion scattering (LEIS) measurements have confirmed that the as-deposited surfaces are composed exclusively of phosphorus and copper (Figure S2), i.e., the presence of impurities, if any, are below the detection limits of LEIS.

**Figure 1:**
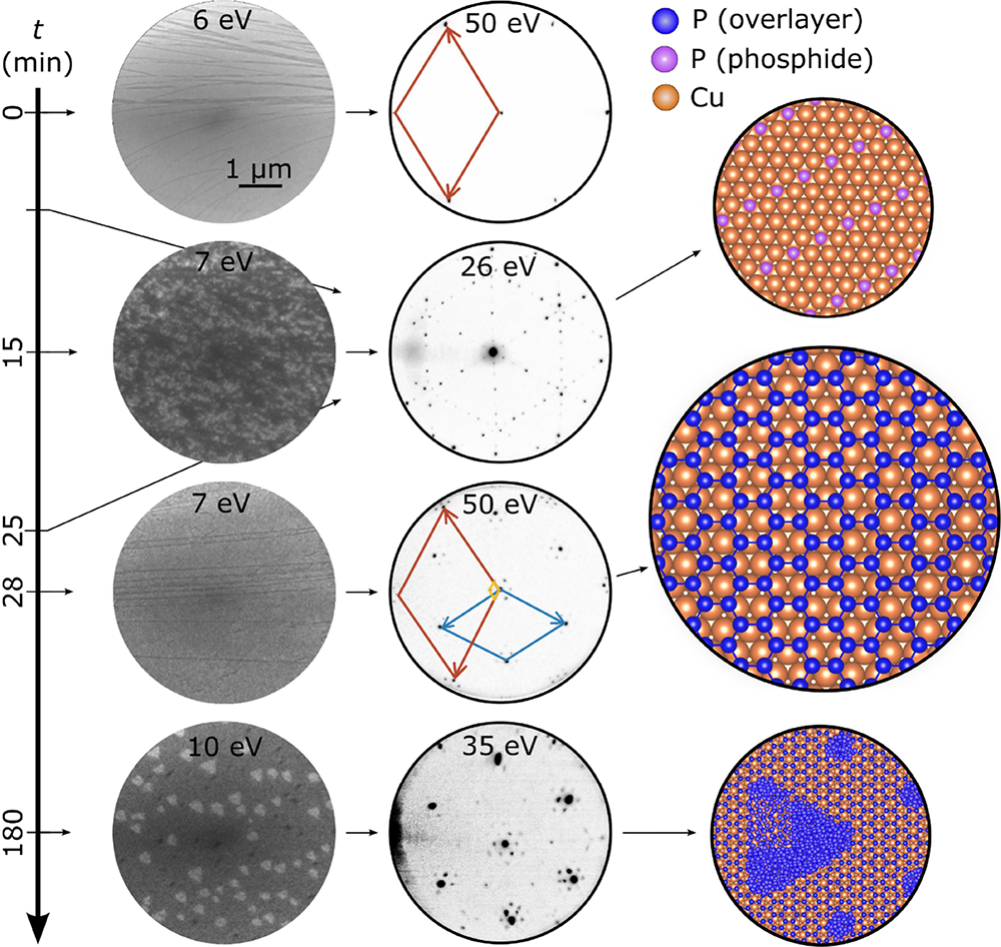
Overview of the representative phase transformations observed during the phosphorus deposition on Cu(111). The first and second columns are in situ bright-field low-energy electron microscopy (LEEM) images (5 μm field of view) and low-energy electron diffraction (LEED) patterns, respectively, acquired as a function of time *t* during exposure to phosphorus (from a GaP effusion cell) at a deposition rate of 0.038 monolayers (ML)/min on single-crystalline Cu(111) at temperature *T* = 450 K. The third column shows schematics of the surface structures determined from the experimental data. At time *t* = 0, the Cu(111) surface is clean, after which the surface is continuously exposed to phosphorus for 180 min. Starting at *t* ≈ 15 min, the Cu(111) surface is covered with a new phase, whose contrast and structure are shown in the LEEM and LEED data in the second row from the top. A detailed structural analysis of this phase is presented in Figure 2. Despite subtle variations, the LEED pattern appears unchanged until around 25 min. Then, the pattern suddenly changes (within a minute) and quickly evolves into a well-defined moiré pattern; see the LEED data in the third row and Figure 3. Further deposition results in the formation of a hexagonal array of phosphorus clusters, followed by phosphorus islands. That is accompanied by a very slow change in the diffraction pattern, as seen in the fourth row LEEM and LEED data. The electron energies for bright-field imaging are chosen to provide a distinct contrast among the different surface features: Cu(111) surface steps appear as dark lines, different phosphide superstructures, triangular and elliptical islands on top of the two-dimensional (2D) phosphorene layer appear brighter and darker gray compared to the surface. The LEED patterns were obtained with the electron beam perpendicular to the sample surface resulting in image distortions at the edges and, hence, cannot be used for quantitative determination of the structural parameters. For accurate measurements, a different experimental geometry was used (see Figure 4).

Figure 1 shows a typical sequence of bright-field LEEM images and LEED patterns acquired during the deposition of phosphorus onto the Cu(111) single-crystal held at *T* = 450 K along with schematics of the compositional and structural changes occurring at the surface. The top row in Figure 1 shows a representative LEEM image and the corresponding LEED pattern obtained from bare Cu(111). (Here *t* = 0 refers to the time at which P deposition was initiated.) Atomically smooth terraces, monatomic steps, and step bunches can be seen in the LEEM image and 6-fold symmetric spots in the LEED pattern, all characteristics of a clean, unreconstructed Cu(111)-1 × 1 surface. With the onset of P deposition, the in situ LEEM image shows the surface covered with brighter and darker areas (domains), and LEED data reveal the emergence of new, qualitatively similar patterns as a function of *t*; one such a typical LEED pattern obtained at *t* = 15 min is shown in Figure 1, second row from the top. At around *t* = 26 min, when the nominal amount of deposited P reaches 1 ML, the LEEM bright-field image shows a uniform surface with substrate steps visible; the initial LEED pattern disappears and new six-fold symmetric spots appear (see Figure 1, third row from the top). Each of these spots is surrounded by six satellite spots, indicative of a higher-order periodicity (a moiré) formation. After prolonged deposition, bright triangular-shaped islands can be seen in the LEEM image; more spots appear in addition to the moiré pattern in the LEED (see Figure 1, bottom row). We suggest and justify below that the deposition of phosphorus on Cu(111) results first in the formation of a transient phosphide phase, which subsequently transforms into a phosphorene monolayer, followed by the nucleation and growth of 2D and 3D islands. We will describe in detail below each of the structural transitions observed in the LEED patterns.

### Initial Growth Phase

**Figure 2:**
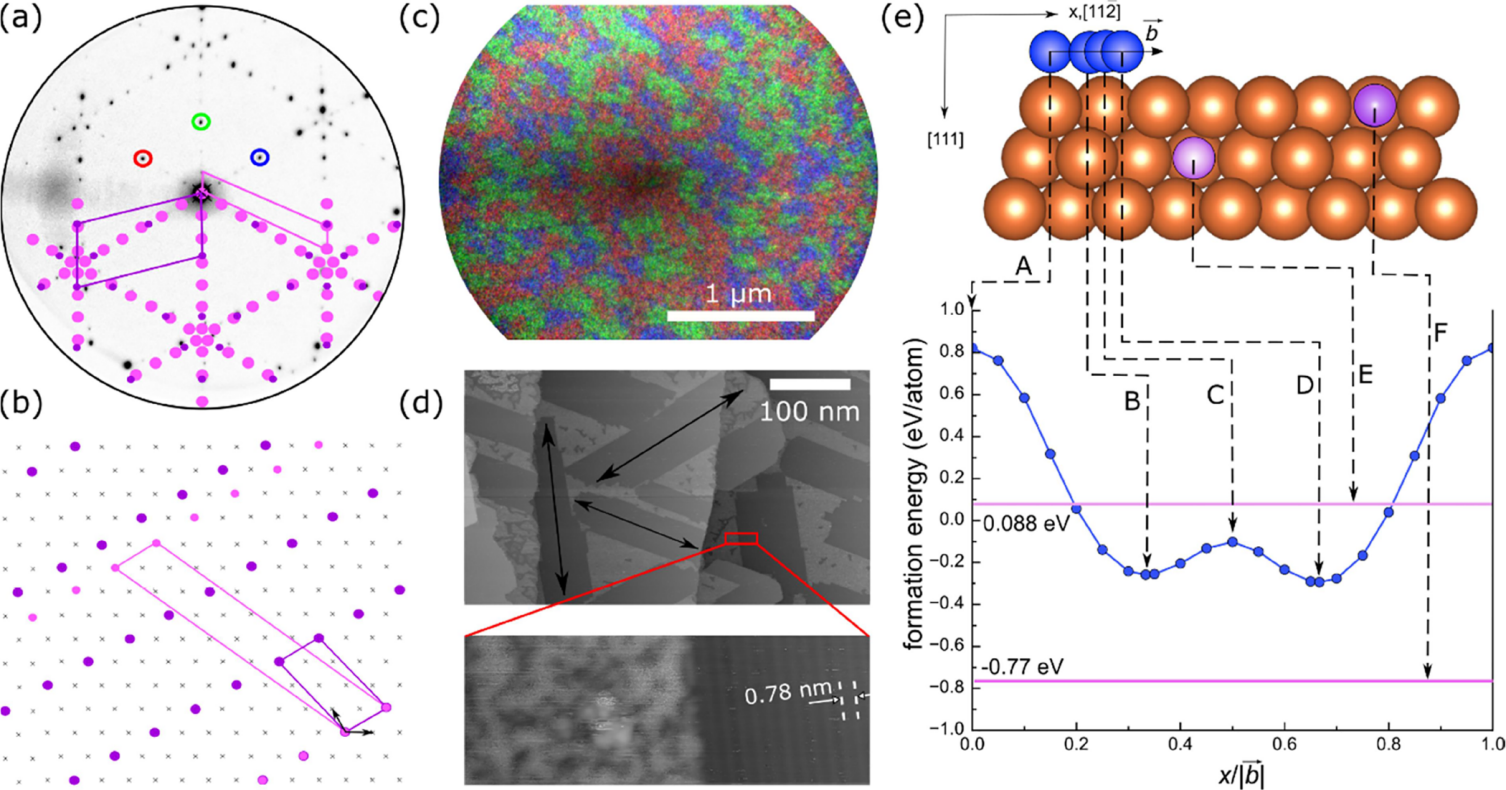
Characterization of the first copper phosphide structure and density functional theory (DFT) modeling. (a) Typical LEED pattern (*E* = 26 eV) obtained from the Cu(111) sample at *T* = 450 K and *t* = 15 min during the P deposition at 0.038 ML/min. (b) Schematic of the atomic arrangement. In (a) and (b), violet and pink colors denote P atoms forming different superstructures on Cu(111). In (b), the first layer Cu atoms are represented with the symbol x, and the primitive cell of the Cu(111) substrate is marked with black arrows. (c) Composition of dark-field LEEM images (*E* = 26 eV) corresponding to three diffraction spots in the LEED pattern in (a). Blue, red, and green colors in the image highlight three rotational domains, associated with diffraction spots marked by blue, red, and green circles, respectively, in (a). (d) Representative scanning tunneling microscopy (STM) images obtained from the same sample as in (a) after cooling to room-temperature using tunneling bias *V*_T_ = 2.22 V and tunneling current *I*_T_ = 50 pA (top) and *V*_T_ = 2.26 V and *I*_T_ = 50 pA (bottom). The black arrows indicate the orientation of surface stripes seen at higher magnification. Additionally, the higher resolution bottom STM image reveals spatial periodicity of ∼0.78 nm. (e) Plot showing DFT-calculated formation energy per phosphorus atom as a function of position *x* parallel to the vector  = [112̅] on Cu(111). Schematic showing a cross-sectional view of the first three layers of Cu(111) with orange, blue, and purple colors denoting Cu, surface adsorbed P, and embedded P atoms, respectively. The lattice sites A–F are as marked in the schematic, and the corresponding formation energies are identified in the plot. A–D are adatoms in top (A), hollow (B,D) and bridge positions (C), and E and F are phosphorus atoms embedded into the copper substrate (first layer–F, second layer–E). Additional top view of the atomic slab is in Figure S4.

First, we focus on the changes in the surface structure observed in the diffraction patterns during the early stages (i.e., *t* ≤ 15 min) of the phosphorus deposition on bare Cu(111) at *T* = 450 K. Movie S1 in the SI shows bright-field LEEM observations during the P deposition on Cu(111). LEEM data reveal step motion and the formation of new steps on the copper surface at *t* between 10 and 18 min. At initial times during the deposition, spots in the LEED patterns are barely visible, diffuse, and of weak intensities (beginning with the one in Figure S3). Sharper, higher intensity, spots appear after approximately 15 min (see Figures 1 and 2a). The LEED pattern shown in Figure 2a is due to two superstructures:  and . The real space model of the superstructures in Figure 2b shows that both are made of rows of P atoms (violet and pink). The diffraction pattern reflects the presence of three rotational domains on the substrate; areal coverages of the three domains, colored red, green, and blue, can be seen in the composite dark-field LEEM image in Figure 2c. Individual domains are probably even smaller than viewed by dark-field imaging (as deduced from varying intensities within individual dark-field images) and do not follow any substrate morphology. Nevertheless, the domains cover the entire surface. In order to determine the local structure of these domains, we used the STM. Figure 2d shows typical lower and higher magnification STM images obtained from the same sample as in Figure 2a,c after being cooled to room temperature. The top STM image in Figure 2d shows darker gray domains along three orientations (highlighted by black arrows) separated by lighter gray features. The higher magnification STM image in the bottom panel of Figure 2d is acquired from a region across the interface between a domain and a lighter gray region, which appears disordered (more on this later). Within the domain, periodically distributed brighter stripes can be seen. The distance between the stripes is ∼0.78 nm, which is equal to the distance between the atomic rows of the  superstructure. The surface height profiles (not shown) of these stripes reveal picometer-scale protrusions, suggesting that the stripes are not formed by a new layer of adatoms but rather due to rows of phosphorus atoms embedded in the Cu(111) surface.

We also carried out density functional theory (DFT) calculations to assess the relative energetics associated with P adatoms and with intercalated P atoms. The DFT calculations show that an exchange of the copper and phosphorus atoms in the first copper layer is plausible, which could yield the surface phosphide phase. First, P adatom was traced along the path of the vector  (see Figure 2e) while calculating the formation energy per phosphorus atom. Next, the phosphorus atom was inserted instead of copper in the first and second layers. From the plot in Figure 2e, clearly replacing a Cu atom in the first layer with a P atom is energetically preferred.

### Transition to a Moiré Phase

**Figure 3:**
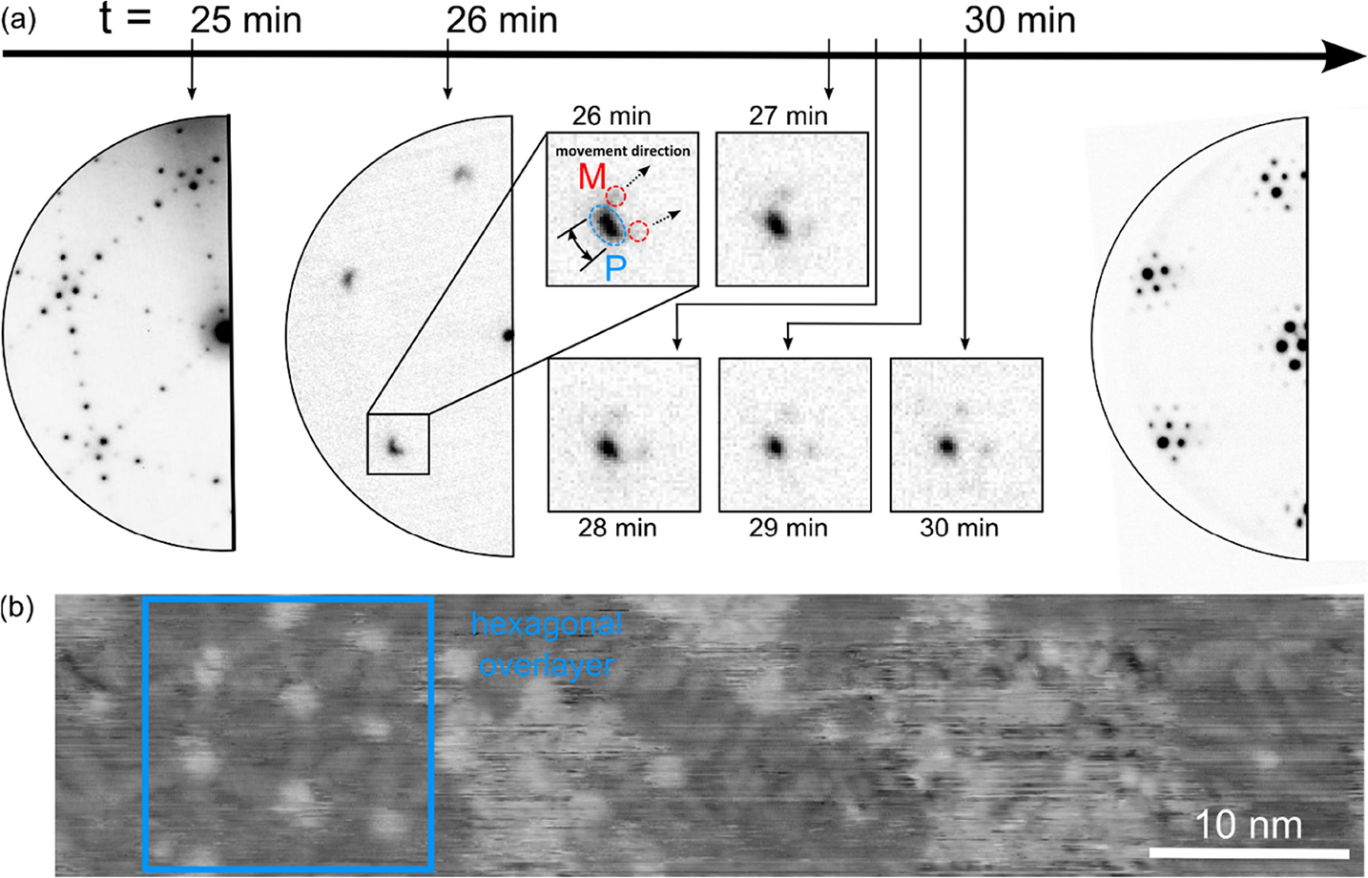
(a) Phase transformations of P/Cu(111) captured in the reciprocal space. In situ LEED patterns (*E* = 26 eV) from the Cu(111) sample as a function of the deposition time *t* at *T* = 450 K after ≈25 min of P deposition at 0.038 ML/min. Within a minute, a sudden change is observed in the LEED pattern: 6-fold symmetric single, diffuse spots appear in the place of the previous multiple spot patterns due to the phosphide phase. One such spot is labeled P and encircled in blue in the higher magnification image of the pattern at *t* = 26 min (see the inset). The spot width, a measure of its sharpness, is highlighted by the black double arrow. With increasing *t*, diffraction spots (highlighted by red dashed circles) of the moiré pattern (labeled M) become visible, moving away from the P spot as indicated by the two arrows. Concurrently, the P spot sharpen as seen in the inset at *t* = 30 min. At *t* > 30 min, LEED shows a sharp and stable spot pattern. Real-time dark- and bright-field LEEM images of the phase transition are shown in SI (Movies S2 and S3). (b) Representative STM image (*V*_T_ = −1.6 V and *I*_T_ = 53 pA) of the Cu(111) sample obtained at room-temperature after the P deposition for *t* ≈ 390 min at *T* = 450 K. The deposition rate in this experiment was ∼0.002 ML/min.

We now focus on structural changes occurring after approximately 1 ML of P deposition, which corresponds to *t* = 26 min in Figure 1 data. For 15 ≤ *t* ≤ 25 min, the LEED patterns (e.g., Figure 2a) vary little as more  phosphide superstructure domains form. This result suggests that there are no changes to the phosphide phase (e.g., formation of (√3 × √3) superstructure or other higher density phosphide) and is supported by our DFT calculations (see Figure S5). After approximately 25 min of deposition, within the next 60 s, the initial LEED pattern disappears and six new diffraction spots with a hexagonal arrangement emerge (see Figure 3a). (The phase change is also documented in situ in dark-field and bright-field LEEM videos, Movies S2 and S3, respectively.) The diffraction spots are initially diffuse and elongated in both the radial and azimuthal directions, suggesting the existence of rotational disorder and strain within the hexagonal layer, respectively. However, the pattern evolves quickly with continued deposition; the spots sharpen, and satellite spots appear. The latter is the first order moiré spots, a signature of two mismatched/misoriented lattices superposed on one another. Based on this observation, we conclude that the observed phase transition results in the formation of a new atomically ordered layer. It is interesting to note that the transition from the phosphide phase to a hexagonal layer occurs relatively fast (within 60 s) compared with the deposition rate (0.038 ML in 60 s) of P atoms. The number of atoms required to form a new overlayer of P, i.e., on top of the phosphide superstructure is 1.35 × 10^15^ cm^–2^ (as calculated for blue phosphorene with the lattice parameter 4.14 Å, see further), much higher than the 6.72 × 10^13^ atoms/cm^2^ deposited within the 60 s transition time. Furthermore, after the transition, the LEED patterns show only two sets of spots, associated with Cu(111) and the hexagonal layer. That is, there is no evidence of the presence of the previous phosphide superstructures. Therefore, we rule out the possibility of a new overlayer lying on the existing phosphide surface and suggest that the hexagonal P overlayer has been formed at the expense of the phosphide superstructure directly on Cu(111). We note here that the phosphide phase is not the only source of P atoms for the transformation, which is further explained in the Discussion section.

In order to capture the phase change with a better time resolution, we carried out another growth experiment using much lower phosphorus flux (yielding 1 ML in 6 h), stopped deposition as soon as the hexagonal pattern appeared in LEED, and cooled the sample to room temperature for STM characterization. The STM image in Figure 3b is acquired from such a sample with ∼0.78 ML of P coverage. The image shows disordered as well as highly ordered areas. The hexagonal symmetry in the region bounded by a blue square in Figure 3b has been observed previously and identified as a flat blue phosphorene overlayer (13). We provide supporting LEED evidence below.

### Moiré Phase

**Figure 4:**
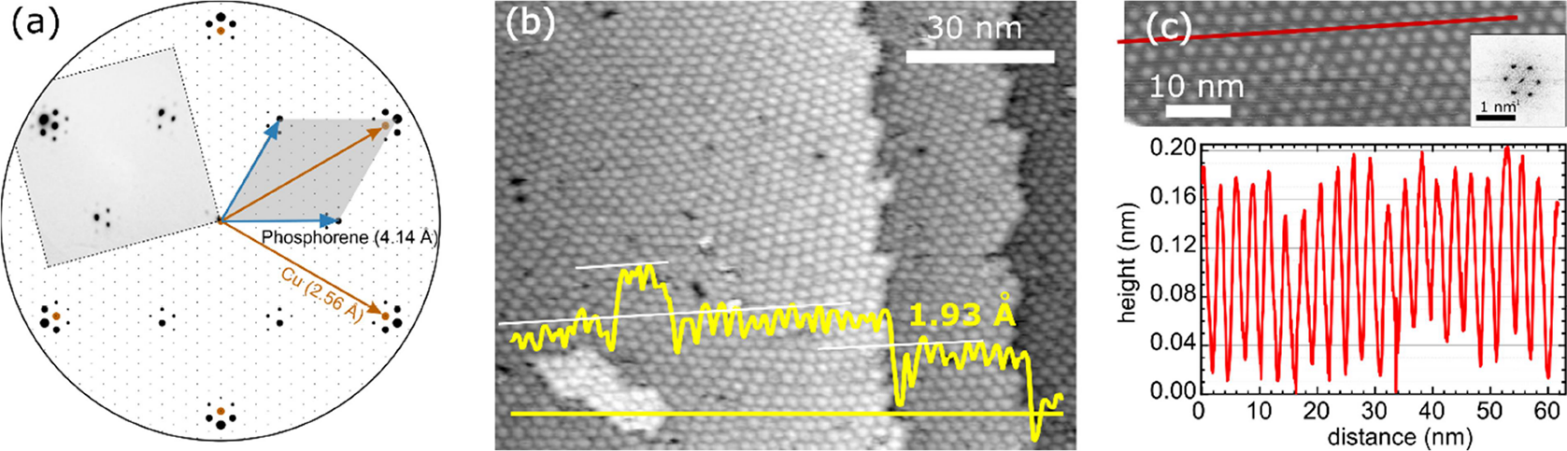
Surface structure of the moiré phase. (a) Composite of the calculated diffraction pattern superposed with the experimental LEED data (the gray colored square inset) acquired using *E* = 16 eV in an off-axis geometry (electron beam was tilted with respect to the sample to move the phosphorene spots at the periphery closer to the center of the Ewald sphere) to mitigate image distortions from the Cu(111) sample at *T* = 450 K after ≈30 min of P deposition at 0.038 ML/min. The orange and blue arrows highlight spots associated with Cu(111) and BlueP. (b,c) Typical room-temperature STM images of the same sample obtained using (b) *V*_T_ = 2.00 V, *I*_T_ = 200 pA and (c) *V*_T_ = −2.00 V, *I*_T_ = 52 pA showing highly periodic cluster island morphology, laying on top of BlueP. The yellow and red curves are surface height profiles along the yellow and red lines, respectively. The surface corrugations are periodic with ∼2.8 nm peak–peak distance and 0.15 nm amplitude; hexagonal arrangement of the cluster islands is confirmed by the Fourier transform, the inset in (c), of a typical, larger STM image (not shown).

With the continued P deposition after the observation of the moiré pattern, the moiré spots move radially outward relative to the primary spots, suggestive of a slight change in the relative orientation of the overlayer with respect to the substrate. Finally, after approximately 30 min of deposition a sharp and stable moiré pattern is established as in Figure 4a. The emergence of the new structural phase is further confirmed by XPS data (Figure S6), which shows that the P 2p peak shifts to higher binding energy by ∼0.17 eV with the transition from the phosphide phase to the hexagonal overlayer.

Figure 4a shows a representative LEED pattern with higher order moiré spots, indicative of a long-range order of the emergent overlayer (we have found that the hexagonal overlayers can be grown at *T* up to 660 K with better quality moiré patterns at higher *T*). The analysis of the spot positions in Figure 4a, assuming a Cu(111) in-plane lattice parameter of 2.56 Å, yields an overlayer unit cell size of 4.14 Å and a moiré periodicity of 35.8 Å. The overlayer lattice parameter is ∼24% larger than that of freestanding phosphorene (3.33 Å),^6^ however, similarly large lattice constants (4.10 Å in ref (13) and 4.20 Å in ref (20)) have been previously reported for BlueP.

STM characterization of the same sample provides a more comprehensive picture of the surface after the phase transition to the moiré phase. Typical STM images (Figure 4b,c) show a spatially periodic array of clusters with a uniform contrast on the terraces, on top of Cu(111) islands, and across the surface steps. The substrate step heights determined from STM images acquired over a wide range of tunneling biases from −3 to +2 V are (1.93 ± 0.10) Å; this value is, within the measurement uncertainties, the same as a step height of (2.03 ± 0.04) Å obtained for pure Cu(111) prior to the P depositions, and is comparable to a step height of 2.08 Å for bulk Cu reported by others.^22^ The moiré beating frequencies and hexagonal overlayer (as previously seen in Figure 3b) are not visible in the STM image, presumably because the contrast is relatively weak compared to the brightness of the clusters that lay on top of the overlayer. Nevertheless, the observation of a continuous and conformal overlayer (deduced from the well-arranged array of clusters on top) is considered a characteristic of carpet growth of vdW layers whose orientations relative to the substrate give rise to periodic moirés with high corrugation amplitudes.^23^ That is, the STM results provide further (yet indirect) evidence for the formation of phosphorene, a hexagonal overlayer on Cu(111). The clusters observed in STM lay on top of the phosphorene. From the Fourier transforms (such as the one in Figure 4c inset) of STM images, an average spatial periodicity of the clusters is determined to be (29.0 ± 1.9) Å and the height is up to 2 Å at maximum for long deposition times (measured heights being independent of STM tunneling bias, ranging from −3 to +2 V).

### Island Phase

**Figure 5:**
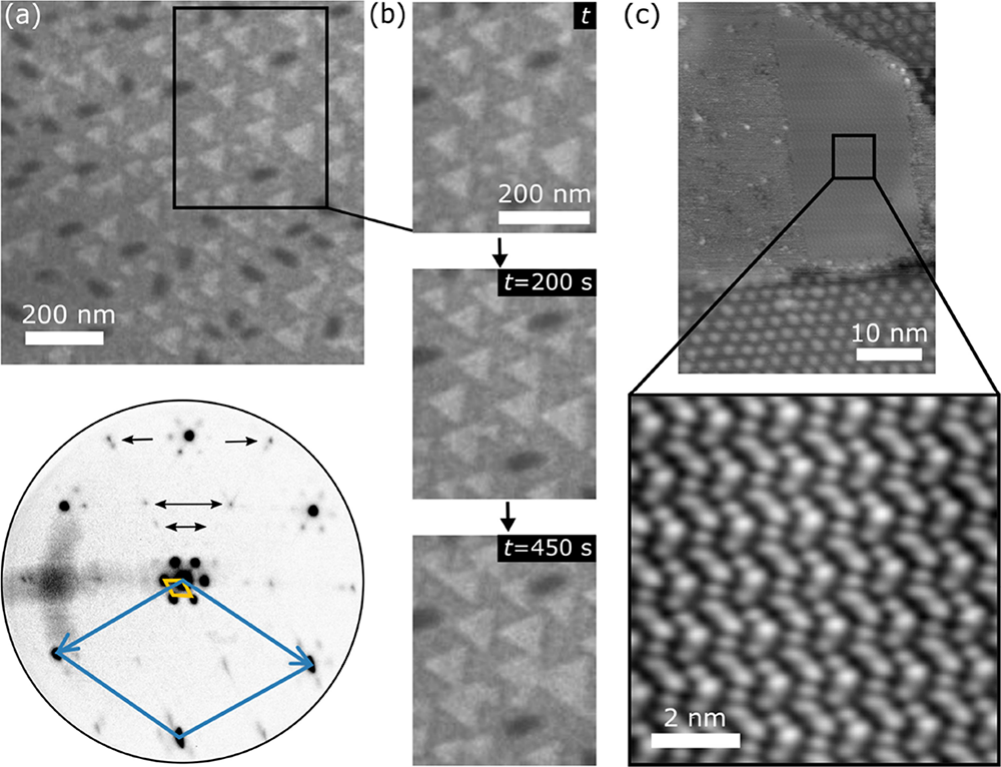
Triangular and elliptical island growth and analysis. (a) Typical bright-field LEEM image (*E* = 7 eV) and LEED data (*E* = 26 eV) acquired from the Cu(111) sample at *T* = 450 K and *t* = 105 min during the P deposition at 0.038 ML/min. In the LEED pattern, the black arrows mark the spots that move with increasing *E*. (b) Series of LEEM images showing the growth of islands with increasing time *t* during the P-deposition. Longer measurement sequences showing energy-dependent changes in LEED and morphological changes in LEEM are presented as Movies S4 and S5, respectively, in SI. The electron energies are chosen to provide a distinct contrast of the substrate (medium gray), triangular islands (bright), and elliptical islands (dark). (c) Typical STM images (*V*_T_ = 2.20 V and *I*_T_ = 50 pA) of a triangular island (top) and higher magnification view (bottom) of its surface, a portion of which exhibits a zigzag pattern, obtained from a Cu(111) sample after 240 min of the P deposition with *T* = 505 K. The triangular island is surrounded by a hexagonal cluster array of the moiré phase.

Continued deposition of P at *T* < 550 K on the moiré surface results in the nucleation and growth of larger islands. Figure 5a is a representative bright-field LEEM image, obtained from the same sample as in Figures 1–4 after *t* = 105 min at *T* = 450 K, showing two types of islands, elliptical and triangular shaped, that appear in darker and brighter gray, respectively. The LEED pattern in Figure 5a bottom panel, obtained during this measurement sequence, reveals additional diffraction spots that move with incident electron energy *E* (see Movie S4 in SI), indicative of the 3D nature of the islands. (In situ LEED data obtained during the deposition of P at *T* > 550 K shows only moiré patterns without the extra spots, which we attribute to the absence of island growth at this *T*.) Figure 5b and Movie S5 in the SI show that the islands grow and coalesce with increasing time. STM images (see Figure S7a) acquired from the same sample show that elliptical islands are several nanometers tall and markedly three-dimensional. Higher-resolution STM images (not shown) of elliptical islands did not reveal any atomic-scale ordering, presumably because these islands are bound by vicinal surfaces. In comparison, the triangular islands are relatively short (Figures 5c and S5b) with atomically flat stepped surfaces and heights comparable (or larger) to those of the clusters in a hexagonal array (see Figure 4c) and rarely over a nanometer. Interestingly, the triangular islands exhibit height variations in 0.5 Å steps (Figure S7c). These steps often bunch, forming 2.0–2.5 Å, macroscopic height step bunches (Figure S7d); such atomically corrugated surfaces are associated with unstable facets.^24^ Importantly, STM images of triangular islands (Figures 5c and S7b) deposited at *T* ∼ 505 K reveal a zigzag pattern at the island surface. However, such a structure was not observed in the triangular islands grown at *T* below 473 K.

## Discussion

Our in situ LEEM and LEED data obtained during the P deposition present direct evidence for two previously unreported phenomena: (1) the growth of phosphide superstructures and subsequent transformation to BlueP hexagonal overlayers on Cu(111) and (2) the growth of phosphorus multilayer islands on top of the hexagonal overlayer. As phosphorus is expected to interact strongly with Cu(111), the formation of phosphides on Cu(111) rather than on BlueP layers seems to be plausible. However, a recent theoretical study^21^ has predicted otherwise and BlueP-like layers have been already experimentally demonstrated.^12,13^ It is in this context that the conversion of a phosphide phase to a hexagonal phosphorene monolayer is intriguing and rather unconventional, compared to the nucleation and growth of 2D layers.^25^ Furthermore, the observation of multilayer islands on top of the phosphorene layer is somewhat surprising because previous studies have ruled out such a possibility. To consistently explain all of our observations, we hypothesize that there exists a critical concentration of P atoms below (above) which phosphide (phosphorene) is stable on Cu(111). It is then possible that during the P deposition, with the increasing P adatom concentration, a rapid phase change can occur resulting in phosphorene formation as proposed recently.^5^ The key requirement therefore is to facilitate surface accumulation of phosphorus, for which the deposition of P monomers is preferred, due to their relatively strong bonding with Cu.^5^ However, the exact nature of the depositing species depends on the phosphorus source; for example, with black phosphorus as the evaporation source, P_4_ clusters are produced.^26^ In our experiments, the decomposition of GaP results in predominantly P_2_ dimers and a smaller portion of P monomers (see Figure S1). DFT calculations predict that the absolute value of the adsorption energy of P_2_ is higher than that of P_4_ on Cu(111) (Figure S8). It is also seen that on the Cu(111) surface, P_4_ clusters dissociate more easily than P_2_ into monomers. We realize that the DFT calculations may not be applicable to the experimental conditions (e.g., elevated temperature and the presence of the P flux). Nevertheless, the DFT results could help us understand the role of the phosphorus source on the differences in the resulting structures^27,28^ and in controlling the growth modes of phosphorene-like layers reported by others,^12,13^ being distinct from our observations. In our experiments, it is likely that the deposition flux of predominant P_2_ dimers with only a smaller portion of P monomers may not be sufficient to provide the P supersaturation required for the phosphorene nuclei formation, and hence, the phosphide is the first phase being formed under these conditions. The phosphide formation, however, is limited to the outermost surface layer as the diffusion of P adatoms into deeper subsurface layers is not energetically favorable (Figure 2e). With the continued deposition, as the P adatom concentration on the surface builds up parallel to the phosphide, the phosphide phase becomes unstable and a phosphorene layer formation is favored, resulting in a rapid phase transition observed in our experiments. The transformation includes the P atoms from the phosphide as well as P adatoms accumulated on the surface (Figure 2d). The diffraction data confirm the formation of a hexagonal monolayer. The STM image acquired during the phase transformation (Figure 3b) appears to be identical to the one reported by Song et al.^13^ The resulting phosphorene is hexagonal with an in-plane lattice constant of 4.14 Å. Although this value is significantly larger (>21%) than that of freestanding phosphorene, similarly large lattice constants (e.g., 4.10 Å in ref (13) and 4.20 Å in ref (20)) have been previously reported.

On the contrary, Kaddar et al. (ref (12)) reported phosphorene with a lattice constant of 3.4 Å, using black phosphorus as the evaporation source. In this case, P_4_ clusters are dominant in the deposition flux. Compared with P_2_ dimers, P_4_ decomposes relatively easily to P monomers upon deposition on the surface. It is plausible that the presence of monomers rather than dimers leads to differences in surface adatom mobilities, attachment/detachment rates, and hence the critical supersaturation required to nucleate phosphorene. Presumably, the deposition of P monomers favors the formation of buckled phosphorene with the lattice constant comparable to the theoretically predicted value.^12^ The deposition of P_2_ dimers, as is the case in our experiments, leads to phosphide growth followed by a fast phase transformation resulting in kinetically limited flat phosphorene with a significantly large lattice constant, as observed here and in other works.^13,20^ DFT calculations (Figure S9) of buckled and flat phosphorene structures, with lattice constants 3.3 and 4.1 Å, respectively, reveal that between the two structures only the bond angles change but the bonds do not stretch significantly. The data in Figure S9 also suggest that the interaction between (metastable) flat phosphorene and the substrate is strong to the point that it amounts to biaxial in-plane tension that leads to an atomically flat structure. It should be noted, however, that other effects (e.g., deposition flux), substrate structure (e.g., surface step orientations and step density), surface composition (e.g., unintentional incorporation of foreign elements), and local environment (e.g., residual gas composition, etc.) may also influence the phosphorene nucleation kinetics and growth.

In our case, the phosphide layer thus plays a crucial role during deposition, serving as an additional reservoir of phosphorus atoms for the phase transformation, together with already present adatoms/dimers. The observed phosphide-to-phosphorene transformation is qualitatively similar to the growth of graphene from nickel-carbide on Ni(111).^29^ In the case of C–Ni(111), Lahiri et al. reported that the decomposition of ethylene at lower temperatures results in the formation of a nickel-carbide, which slowly transforms into graphene. At higher temperatures, they observed C dissolution in the Ni bulk rather than surface carbide formation, followed by graphene precipitation. In contrast, we find that the transformation of phosphide to phosphorene is instantaneous. We do not find any evidence of the coexistence of the phosphide and phosphorene. Since the bulk dissolution of P in Cu is not feasible and the phosphide phase is limited to the top surface layer, the P content within the surface phosphide alone is not sufficient to transform into phosphorene. Additional P must be supplied from the vapor phase. Furthermore, as the phosphide transforms into phosphorene, the top substrate layer becomes phosphorus-free. These aspects are distinctively different from the phenomena observed in graphene/Ni(111). Nevertheless, our observations open up the possibility of direct deposition of phosphorene on bulk Cu_3_P substrate, which may have further implications on the growth of other elemental 2D materials.

The STM images of the post-transition phase (Figure 4c) suggest a complex structure, where an array of clusters covers the hexagonal monolayer in a hexagonal pattern (not visible in LEED). This result has been repeatedly reported in previous studies,^12,20^ and thoroughly explained in ref (13): the excess phosphorus atoms nucleate on top of the hexagonal overlayer and follow a self-assembly process into spatially arranged clusters.

The periodicity of the hexagonal array of phosphorus clusters seen in the STM images is 28.7 Å and significantly differs from the moiré lattice parameter (35.6 Å) deduced from the LEED data. The measured periodicity by STM is comparable to the Fermi wavelength (29 Å) of the copper surface. It has been previously reported^30^ that the standing wave patterns in the electron density promote a self-assembly of Cu clusters on Cu(111). The same effect observed in our experiments could suggest that the phosphorene overlayer is "transparent" to the emanating fields, similar to graphene, allowing a remote epitaxy of adlayer clusters.^31^ Nevertheless, this claim requires further investigation.

With the continued deposition, we observe the clusters growing in size but the growth is not self-limiting as previously reported.^12^ Our data show that the extended deposition onto the cluster-covered surfaces results in the formation of low aspect ratio, quasi-2D triangular islands, and relatively higher aspect ratio, 3D elliptical mounds (Figure 5). We attribute the island formation to the attachment kinetics of P_2_ dimers, the dominant depositing species in our experiments, compared to earlier reports. Another explanation may include multilayer phosphorene growth via precipitation or dealloying, similar to graphene on, e.g., nickel or platinum. However, in the Cu–P system, the surface alloy is limited only to the topmost substrate layer and, hence, the available P concentration is not large enough to facilitate large multilayer island formation under the existing layers as is the case in multilayer graphene grown on metals with high C solubility.^32,33^ Finally, we comment on the highly periodic zigzag patterns observed in the STM images of the triangular islands deposited at higher temperatures (*T* ∼ 505 K). While we are not able to assign this particular pattern to BlueP, BlackP, or a moiré due to the superposition of BlueP/BlueP or BlackP/BlueP layers, clearly these islands are crystalline phosphorus structures that appear to be stable on hexagonal phosphorus layers.

## Conclusions

Our in situ LEEM and LEED observations reveal two phenomena: the formation of phosphide superstructures that transition into BlueP hexagonal layers on Cu(111) and the growth of phosphorus multilayer islands atop these layers. While phosphide formation on Cu(111) is expected due to strong P–Cu interactions, our results suggest a critical P concentration beyond which phosphorene becomes stable, leading to a rapid phase transition. The initial phosphide layer likely serves as a phosphorus reservoir, facilitating the high supersaturation needed for phosphorene formation. This transition challenges previous assumptions and underscores the importance of deposition conditions and phosphorus sources in controlling the growth of 2D phosphorene layers. The rapid phase transformation and subsequent island growth highlight the complex kinetics and energetics involved in this system, offering insights into 2D material fabrication on metal substrates. The rapid phase transformation from phosphide to phosphorene suggests a possible mechanism to control the growth of 2D phosphorus layers, which could have significant implications for scalable production.

## Methods/Experimental Section

All the experiments were carried out in an ultrahigh vacuum (UHV) system (base pressure ∼10^–10^ mbar), which houses a SPECS FE-LEEM P90 low-energy electron microscope, SPECS X-ray photoelectron spectroscope (XPS) with the Mg Kα X-ray source and Phoibos 150 spectrometer, SPECS Aarhus 150 scanning tunneling microscope (STM) and high sensitivity IonTof Qtac100 low-energy ion spectrometer (LEIS), among other tools, and allows in vacuo transfer samples under UHV conditions. First, a single-crystal Cu (111) substrate was cleaned by several cycles of 2 keV Ar^+^ bombardment at 10^–5^ mbar at room temperature, followed by annealing at 785 K. Surface morphology, structure, and composition of the as-prepared sample were checked by LEEM, LEED, and XPS, respectively. The substrate temperature *T* during the phosphorus deposition ranged from 420 to 630 K; most of the experiments were performed at 450 K. Phosphorus is generated by preferential evaporation from solid GaP chunks (MBE Komponenten, 6N purity) (for the mass spectrum of evaporated molecules, see Figure S1), directly in the LEEM. Ga atoms are captured by a cap at the end of the crucible, which prevents contamination of Cu(111) with Ga. We measured the pressure increase in the chamber caused by evaporation (analogically to beam flux monitoring utilized in molecular beam epitaxy), which is reproducible and a stable measure of the phosphorus flux. During a typical P deposition experiment, the pressure *p* in the LEEM chamber increased to 1.2 × 10^–9^ mbar with the phosphorus cell at 1080 K. From STM images, we estimated the deposition rate as one monolayer of P atoms (equivalent to a surface concentration of 1.77× 10^15^ P atoms on the Cu(111) surface) per 26 min at *T* = 450 K. It should be noted that the actual deposition rate depends on both the flux of P atoms impinging on the substrate and the sticking coefficient at a given *T*; the higher the substrate temperature the lower the deposition rate.

LEIS, XPS, and STM characterizations were carried out after passive cooling of the sample to room temperature. The electron emission angle in XPS was fixed along the normal path to the surface. Individual XPS spectra were acquired in a high magnification mode, with 25 and 40 eV pass energies for Cu and P, respectively. All of the spectra were acquired with an energy step of 0.1 eV and integrated by utilizing several sweeps. The spectra had not been shifted. The relevant photoelectron peaks were fitted with Voigt functions after Shirley background subtraction. The scanning tunneling microscope was equipped with a KolibriSensor. STM measurements were performed at room temperature in a constant current mode; the sample bias voltages (*V*_T_) and tunneling currents (*I*_T_) were varied and noted with each image. The STM images were not corrected for drift during processing. In LEIS, the focused primary beam of He^+^ ions impacted perpendicular to the surface over a selected area of 2 × 2 mm^2^. The detector collected all the ions scattered at the 145° polar angle into all azimuthal angles (0–2π).

### Details on DFT Calculations

The total energies of all configurations were computed using the ab initio code VASP^34^ employing the projector augmented-wave method.^35^ The exchange and correlation contribution to the energy were evaluated with the help of the generalized gradient approximation parametrized by Perdew et al.^36^ Integrations over the Brillouin zone were performed by using a mesh of equidistantly spaced k-points with a maximum distance of 0.2 Å^–1^. Whenever we optimized the atomic positions, the residual forces on the atoms were relaxed below 0.01 eV/Å. The cutoff energy for the plane-wave basis was set to 500 eV. To simulate the Cu surface, we created a slab of three (111) Cu planes. The two lower planes were kept fixed and only the atoms in the upper (surface) plane were allowed to relax. In order to compare the energy of individual configurations containing *N*_P_ atoms of phosphorus and *N*_Cu_ atoms of Cu, we employed the energy of formation *E*_f_ = , where *E*_tot_, *E*_Cu_, and *E*_P_ are the energies of the particular configuration (a Cu slab with a P atom), a single Cu atom in the fcc bulk, and a single P atom in the blue phosphorene, respectively. *E*_surf_ is the energy of both relaxed upper and fixed bottom surfaces of the Cu slab. The energy of a pure Cu slab can then be expressed as *N*_P_*E*_P_ + *E*_surf_. Note that if one considers the adsorption of P_n_ clusters (with *n* = 2 for dimer and *n* = 4 for tetramer) on the Cu surface, redefining *E*_P_ to be the energy of a single P atom in the considered cluster, the definition of *E*_f_ becomes equivalent to the definition of an adsorption energy (shown in Figure S8).

## Data Availability

The data underlying this study are openly available in Zenodo at doi: 10.5281/zenodo.11192796.

## Supporting Information Available

Residual gas analysis; low energy ion scattering data; additional LEEM and diffraction data; unit cells and crystal slabs used in DFT calculations; XPS comparison of phosphorus phases; additional STM analysis; DFT calculations of adsorption energies of different P clusters and phosphorene cells; and additional references (PDF)
  Surface step movement during the phosphide formation (AVI)
  Dark-field (DF) real-time LEEM imaging of the phosphorene formation (AVI)
  Bright-field real-time LEEM imaging of the phosphorene formation (AVI)
  Energy sweep of LEED patterns from Cu(111) after the prolonged P deposition (AVI)
Bright-field real-time LEEM imaging of the growth of triangular and elliptical islands (AVI)

## Supplementary Material

nn4c11802_si_001.pdf

nn4c11802_si_002.avi

nn4c11802_si_003.avi

nn4c11802_si_004.avi

nn4c11802_si_005.avi

nn4c11802_si_006.avi

## Notes

This manuscript was previously submitted to a preprint server: J.D., F.J., P.P., M.Č., C.V.C., S.P., T.Š., S.K., M.K. Direct observation of structural phase transformations during continuous phosphorus deposition on Cu(111). 2024, arXiv:2408.13090. 10.48550/arXiv.2408.13090 (accessed August 23, 2024).
