# Supplementary material for: Direct Observation of Structural Phase Transformations during Phosphorene Formation on Cu(111)
Source: ACS Nano. 2025 Jan 22;19(4):4289–98. doi: 10.1021/acsnano.4c11802 (PMC11803912; doi:10.1021/acsnano.4c11802)

Supporting information for

Direct observation of structural phase transformations during phosphorene formation on Cu(111)

Jiří David[1], František Jeřábek[1], Pavel Procházka[1,2], Miroslav Černý[1,2], Cristian V. Ciobanu[3], Stanislav Průša[1,2], Tomáš Šikola[1,2], Suneel Kodambaka[4], Miroslav Kolíbal[1,2,*]

[1]Institute of Physical Engineering, Brno University of Technology, Technická 2, 616 69 Brno, Czech Republic

[2]CEITEC BUT, Brno University of Technology, Purkyňova 123, 612 00 Brno, Czech Republic

[3]Department of Mechanical Engineering, Materials Science Program, Colorado School of Mines, Golden, Colorado 80401, United States

[4]Department of Materials Science and Engineering, Virginia Polytechnic Institute and State University, Blacksburg, Virginia 24061, United States

[*]kolibal.m@fme.vutbr.cz

Contents

Fig. S1: Residual gas analysis data in the phosphorous evaporation chamber at base vacuum and with a GaP decomposition cell operated at 1080 K

Fig. S2: Low-energy ion scattering (LEIS) spectrum obtained by 3 keV He^+ ions from Cu(111)-P sample

Fig. S3: *In situ* low-energy electron microscopy and diffraction (LEEM & LEED) data obtained from a Cu(111) bulk crystal at temperature $T = 450$ K during P deposition

Fig. S4: A detailed top-view of the crystal slab utilized in DFT calculations in Fig. 2.

Fig. S5: Examples of computational unit cells employed for calculations of the phosphide formation energy

Fig. S6: X-ray photoelectron spectroscopy (XPS) data comparison of phosphorus phases

Fig. S7: Room-temperature scanning tunneling microscopy (STM) characterization of Cu(111) surfaces with the P island phase

Fig. S8: Density functional theory (DFT) calculations of adsorption energies E_{ads} per atom of different P clusters on the Cu(111) surface

Fig. S9: Density functional theory (DFT) calculations of energy of a 1x1 cell, bond length and bond angle for freestanding BlueP.

Movie S1: Surface step movement during the phosphide formation. Bright-field LEEM movie

Movie S2: Dark-field (DF) real-time LEEM imaging of the phosphorene formation

Movie S3: Bright-field real-time LEEM imaging of the phosphorene formation

Movie S4: Energy sweep of LEED patterns from Cu(111) after the prolonged P deposition

Movie S5: Bright-field real-time LEEM imaging of the growth of triangular and elliptical islands

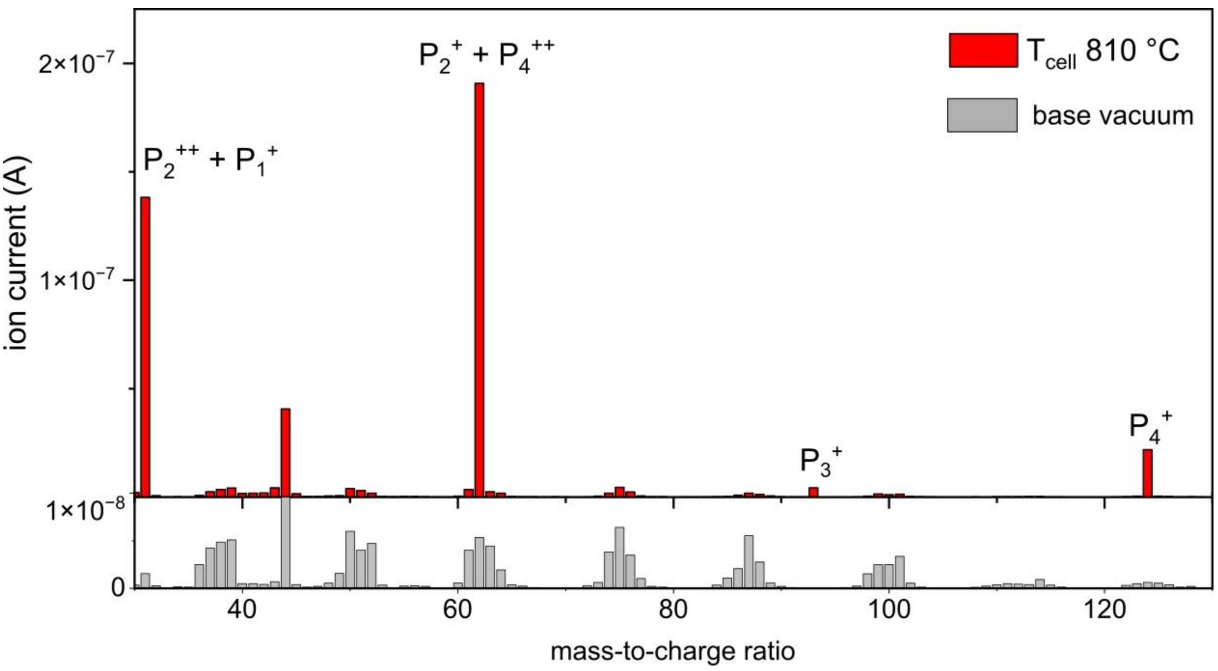

Fig. S1: Residual gas analysis data plotted with the ion current as a function of the mass-to-charge ratio in the phosphorous evaporation chamber at base vacuum (2×10^{-10} mbar, grey) and with a GaP decomposition cell operated at 1080 K (1.24×10^{-9} mbar, red). The increase in the chamber pressure during the thermal decomposition of GaP is due to P_x (x = 1 to 4) clusters generated by the cell. The ratios of ion currents associated with (P_2^+ + P_4^{++}) to P_4^+ and (P_1^+ + P_2^{++}) to P_4^+ are approximately 9:1 and 6:1, indicating that the phosphorus dimers and, to a lesser extent, phosphorus monomers are the majority products of the GaP decomposition. This is in striking contrast to evaporation from black phosphorus, during which P_4 clusters are the dominant species in the vapor.

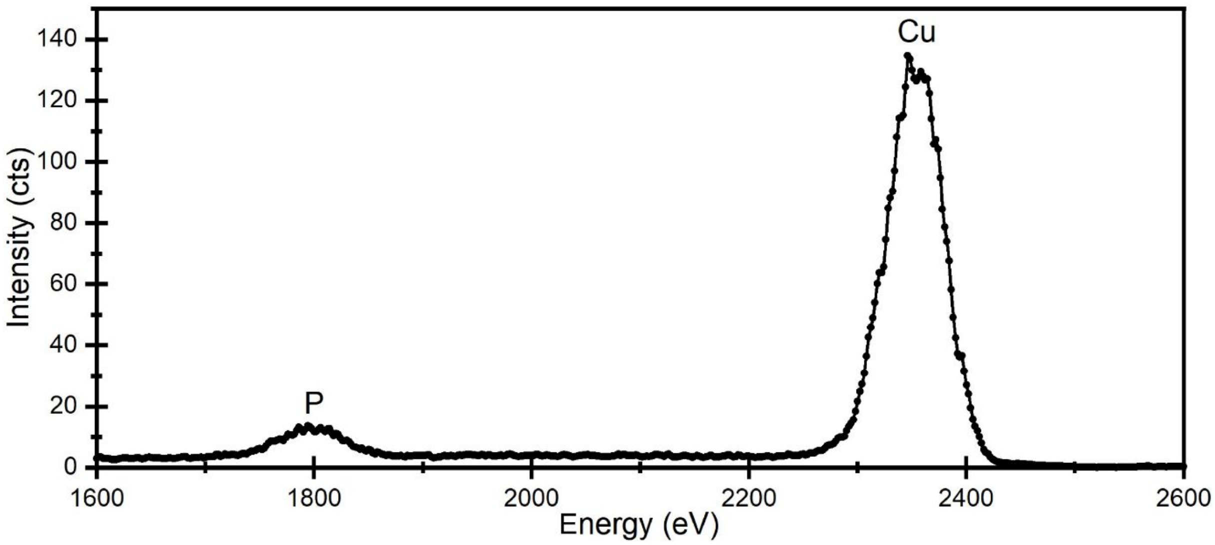

Fig. S2: Low-energy ion scattering (LEIS) spectrum obtained by 3 keV He^+ ions from Cu(111)-P sample with $\begin{pmatrix} 2 & 1 \\ -1 & 3 \end{pmatrix}$ superstructure (the first phosphide phase). The only observable peaks in the data correspond to Cu (2355 eV) and P (1800 eV). The absence of any other peaks, commonly associated with contaminants such as O, S, etc., is indicative of a compositionally pure surface. Similar data can be seen in XPS, but LEIS is more surface sensitive.

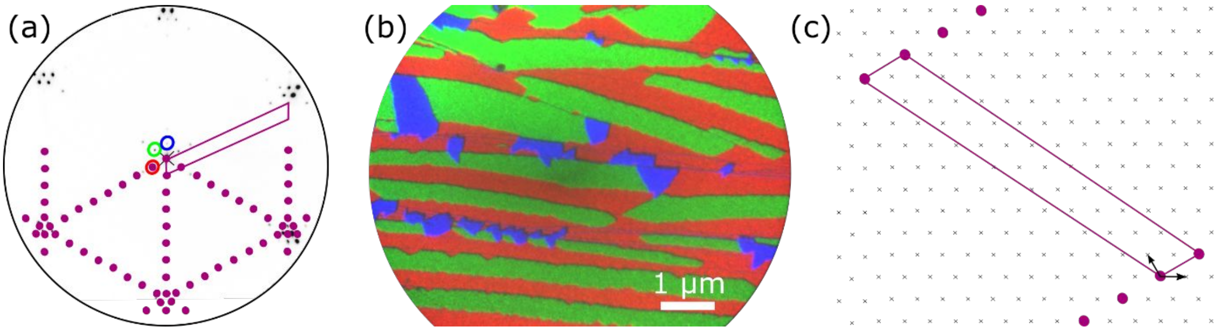

Fig. S3: *In situ* low-energy electron microscopy and diffraction (LEEM & LEED) data obtained from a Cu(111) bulk crystal at temperature $T = 450$ K during P deposition. In this experiment, the P deposition rate is relatively low, compared to that used to obtain data presented in Figs. 1-5, allowing to observe other (transient) phosphide superstructures. (a) Representative LEED pattern obtained with incident electron energy E = 15 eV at deposition time t = 14 min. The blue, red, and green circles highlight three different rotational domains of the copper phosphides with the $\begin{pmatrix} 2 & 1 \\ -7 & 9 \end{pmatrix}$ superstructure identified using spots with violet color. (b) Dark field LEEM image corresponding to the LEED pattern in (a) acquired at t = 15 min. Colors within the image correspond to the three 3 rotational domains identified in (a). (c) Schematic of the atomic arrangement in one domain, noted as the $\begin{pmatrix} 2 & 1 \\ -7 & 9 \end{pmatrix}$ superstructure in the matrix notation. The first layer Cu atoms are marked with crosses. The primitive cell of the Cu(111) substrate is marked by black arrows.

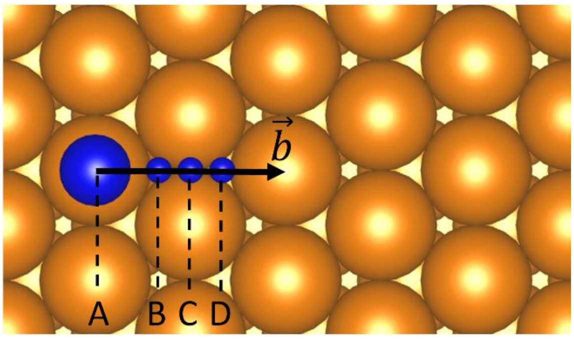

Fig. S4: A detailed top-view of the P adatom (blue sphere) in the top position (denoted A) with hollow (B and D) and bridge (C) positions indicated by the smaller blue circles. Side view of the same system, which was used in the DFT calculations, is shown in Fig. 2(e) in the main manuscript.

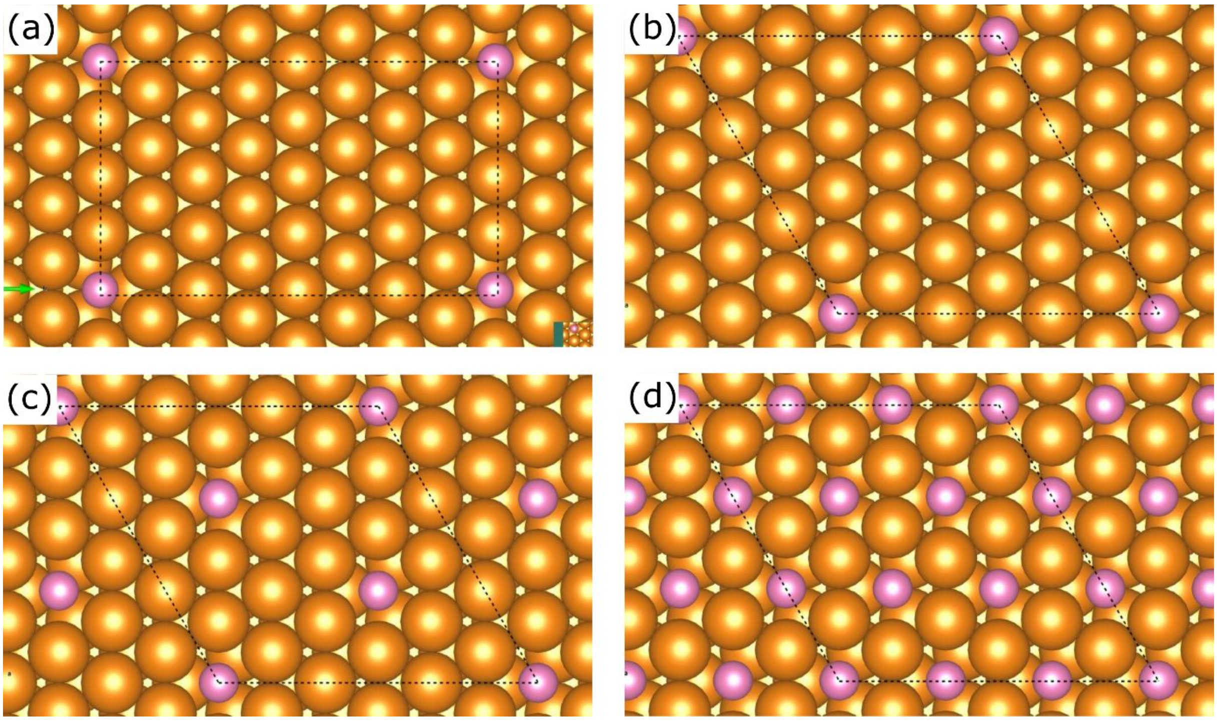

Fig. S5: Examples of computational unit cells employed for calculations of the phosphide formation energy. All the examples show the P atoms (pink spheres) embedded in the surface layer of Cu. Each of the unit cells corresponds to a different P coverage. All the Ef values were scattered within a range of -0.72 eV to -0.78 eV, confirming that embedding the phosphorus in the surface layer is energetically favorable in a relatively wide range of P amount. At the same time, we have not observed any clear trend or correlation of the formation energy with the increasing P concentration within the phosphide. Only for the highest (0.5 ML, not shown here) coverage we have observed substantially higher formation energy, suggesting the instability of a denser phosphide.

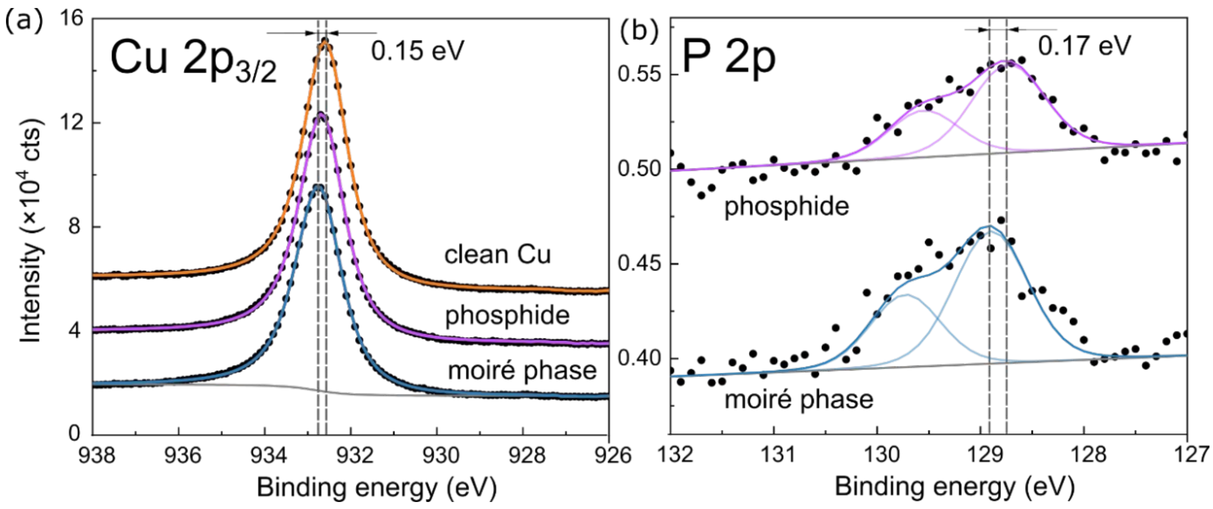

Fig. S6: X-ray photoelectron spectroscopy (XPS) data obtained at room-temperature from a Cu(111) bulk crystal (orange curve), after depositing P at 0.038 ML/min and $T = 450$ K for $t = 15$ min (violet curve), and after the continued P deposition for another $t = 15$ min at the same rate and T (dark blue curve). The samples are transferred *in vacuo* from the deposition chamber to an XPS chamber. With these deposition parameters, we expect the formation of the first of several phosphide structures at $t = 15$ min and moiré phase (phosphorene plus hexagonal array of clusters) at $t = 30$ min. The plots (a) and (b) are XPS data (solid circles) of (a) Cu $2p_{3/2}$ and (b) P 2p peaks. The solid lines are fits to the data using Casa XPS software. Note the shift in the Cu $2p_{3/2}$ peak position to lower binding energy by ~0.15 eV between pure Cu and the first phosphide. Any change in the peak positions between the first phosphide phase and moiré phase are below the resolution limit of this measurement. The P 2p peak shifts by ~0.17 eV between the first phosphide phase and the moiré phase. We have used the following fit parameters in the Casa XPS: For the Cu $2p_{3/2}$ peak, we have applied the Shirley background correction and fit an assymetric Lorentzian-Gaussian function (with the tail spread of the Lorentzian component of 1.3 and 1.4, and 40% Gaussian component) with full width at half maximum (FWHM) of 1.20 eV); the P 2p peak has been fitted as a dublet ($2p_{3/2}$ and $2p_{1/2}$) after the Tougaard U2 background correction using the sum Voight function (with the 30% Gaussian component) and FWHM of 0.8 eV.

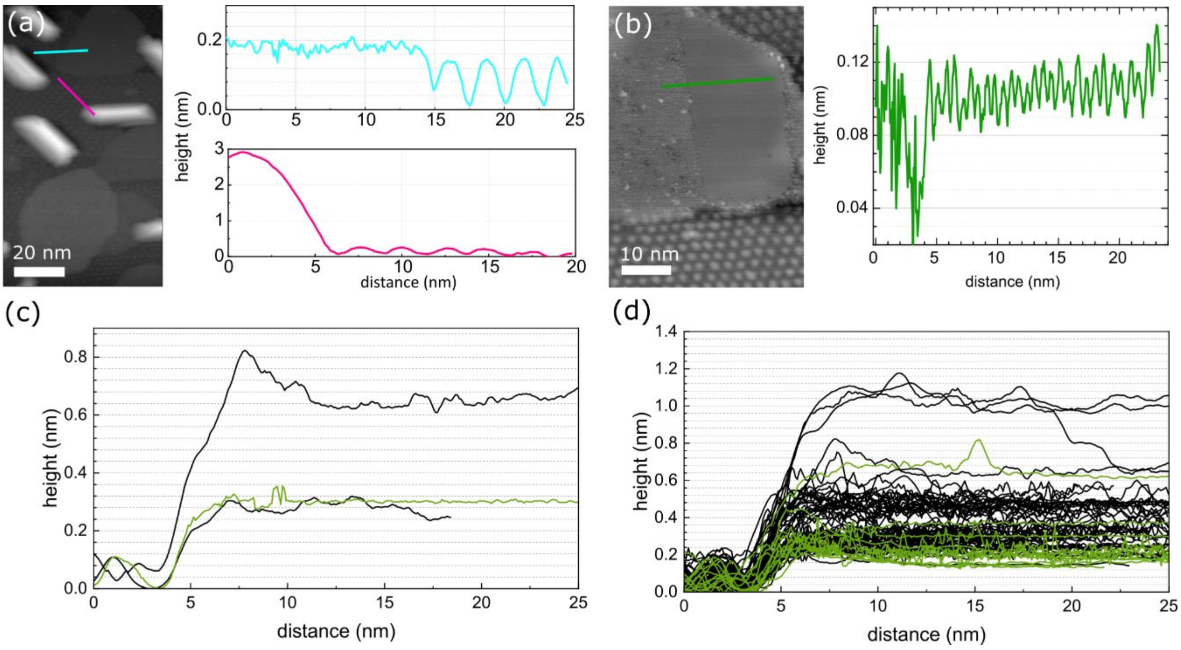

Fig. S7: Room-temperature scanning tunneling microscopy (STM) characterization of Cu(111) surfaces with the P island phase. The STM images are acquired (a) after depositing P at 0.038 ML/min and T = 505 K for t = 240 minutes using tunneling bias V_T = 1.40 V and current I_T = 50 pA and after (b) the P deposition for t = 240 minutes and T = 505 K with V_T = 2.20 V, I_T = 50 pA. After the deposition, the samples are transferred *in vacuo* after the depositions to the STM chamber. The plots associated with the STM image in (a) show surface height profile (purple curve) along the purple line across a typical elliptical island compared to the flat triangular island whose height profile along the cyan line is shown by a cyan curve. The image in (b) shows a zigzag pattern within a triangular island. The associated plot is a surface height profile (green curve) along the green line across the zigzag pattern. The corrugations are periodic with the 0.65 nm peak-to-peak distance and ~0.03 nm amplitude. $V_T > 0$ implies the tip is positively biased with respect to the sample surface, i.e., the images are a measure of filled density of states of the surface. (c,d) Representative surface profiles across triangular island(s) with (green) and without (black) a zigzag pattern. Step bunching (50 pm step height, (c)) is apparent on triangular islands without zigzag pattern.

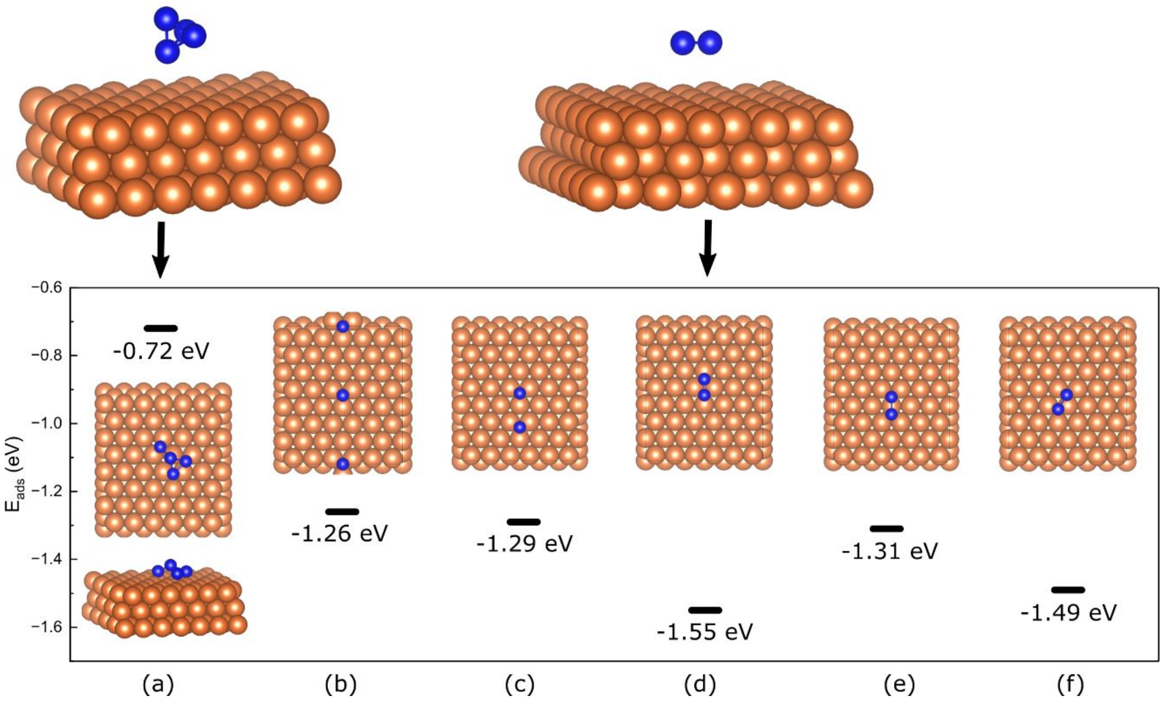

Fig. S8: Density functional theory (DFT) calculations of adsorption energies E_{ads} per atom of different P clusters (blue colored spheres) on the Cu(111) surface (gold colored spheres). (a) Relaxed P_4 cluster upon impingement yields E_{ads} = -0.72 eV. (b,c) P monomers lower their energy as they get close to each other. (d) The most stable configuration that arises from P_2 dimer relaxation on the surface with E_{ads} = -1.55 eV. (e,f) Other P_2 dimer configurations with E_{ads} values lower than those of P monomers, indicating that P_2 dimers are more stable than P monomers.

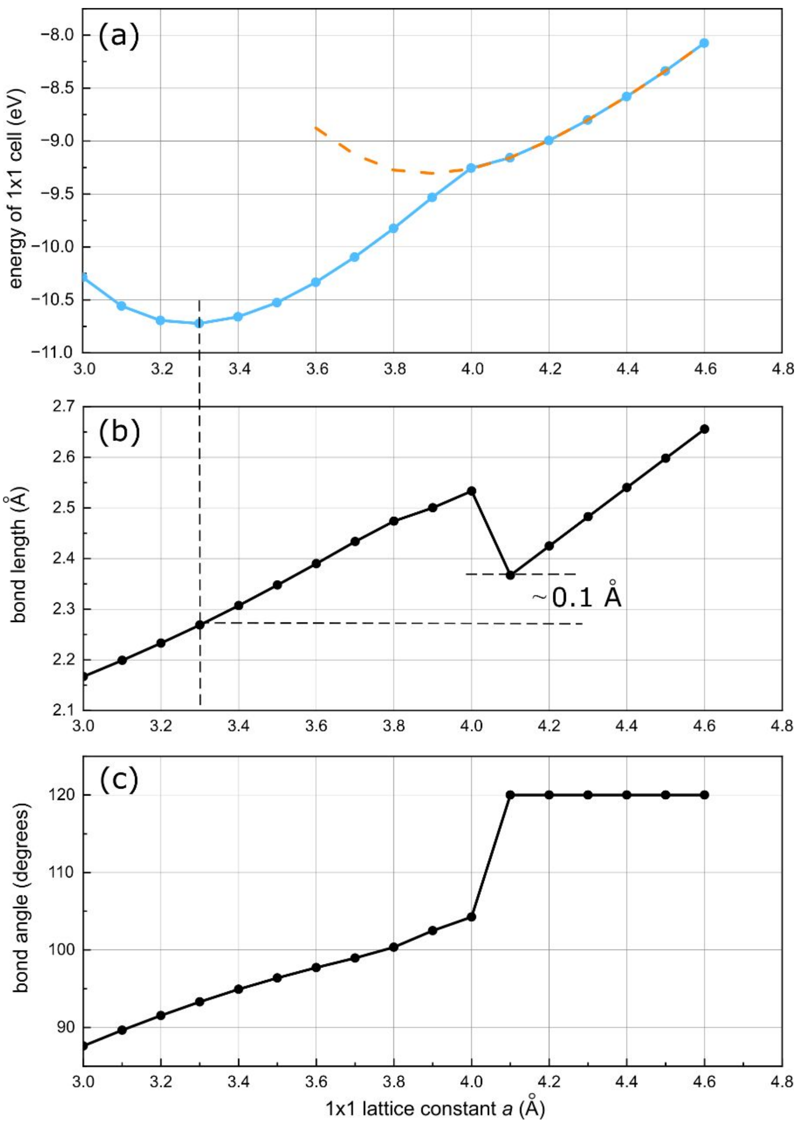

Fig. S9: Density Functional Theory (DFT) calculations of energy of a 1x1 cell, bond length, and bond angle for freestanding BlueP. (a) Solid circles and blue line: Energy of a 1x1 monolayer Blue P unit cell vs. the lattice constant (a) of the unit cell. The energy and structure are obtained from GGA-PBE relaxations starting with the buckled configuration and relaxed at constant supercell dimensions. At 4.1 Å, the monolayer becomes atomically flat. The dashed orange line is the "reverse curve", where the calculations are started with flat structure, beginning with a = 4.6 Å. (b) Bond length and (c) bond angle for the configurations corresponding to solid circles in (a). Note that the bonds do not stretch significantly between the two local minima (3.3 Å and 4.1 Å) in the figure above, only the bond angles change.

Description of Supplementary Movies

Movie S1: Surface step movement during the phosphide formation. Bright-field LEEM movie, part of a longer measurement sequence, shown at 20 frames/s (acquired at 1 frame/s) during the P deposition on Cu(111) held at $T = 433$ K. The images below are part of the LEEM movie. The image width is 1.8 μm; time t in the images corresponds to ~10 minutes after starting the P deposition. The beam conditions are chosen to visualize the Cu(111) surface steps as dark lines with $E = 18$ eV. The movie captures the formation of new steps (marked purple in the figure below) as well as the movement of existing steps (yellow).

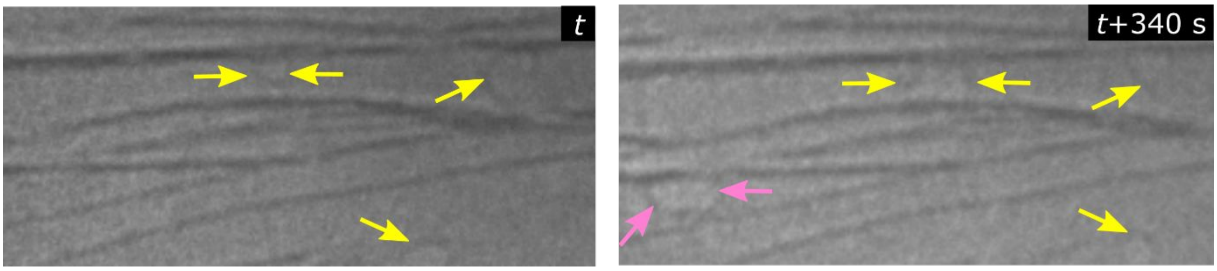

Movie S2: Dark-field (DF) real-time LEEM imaging of the phosphorene formation. The panel below shows a LEED pattern acquired from Cu(111) at T = 423 K using E = 26 eV at $t \approx 25$ minutes during the P deposition. The diffraction spot due to the $\begin{pmatrix} 2 & 1 \\ -1 & 3 \end{pmatrix}$ superstructure (1st phosphide phase) that was used for DF imaging is marked in red on the LEED pattern. LEEM images (width = 3 μm) in the panel are obtained in the DF mode with E = 10 eV and are part of a longer measurement sequence (see Movie S2) showing the transition from a phosphide buffer layer to an hexagonal overlayer. The phosphide domains associated with the chosen diffraction spot appear brighter in contrast in the images. The selected domains fade away and the diffraction spot intensity weakens with the increasing time. Interestingly, the domains also shrink in size.

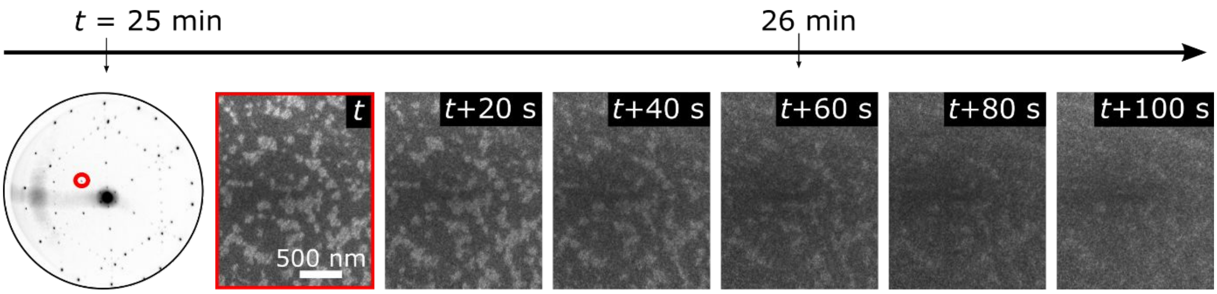

Movie S3: Bright-field real-time LEEM imaging of the phosphorene formation. In this experiment with beam energy E set to 6 eV, the LEEM images (width = 10 μm) are shown at 7 frames/s (acquired at 1 frame/s) during the P deposition with the Cu(111) crystal held at T = 623 K. The higher T is chosen to slow down the transformation of the phosphide phase to phosphorene. In the LEEM images, the phosphide phase appears bright grey at t = 25 minutes. After 212 s, the image reveals areas with two new contrasts due to rotational domains 1 and 2, which appear in place of the original phosphide phase. These two grey levels represent the radial blur of the new spots appearing in the LEED pattern (Fig. 3, spot P) and probably correspond to the two 'chiral' domains observed in Ref. 13. After 420 s, the two rotational domains are replaced by a different grey intensity, which corresponds to the final moiré hexagonal phase.

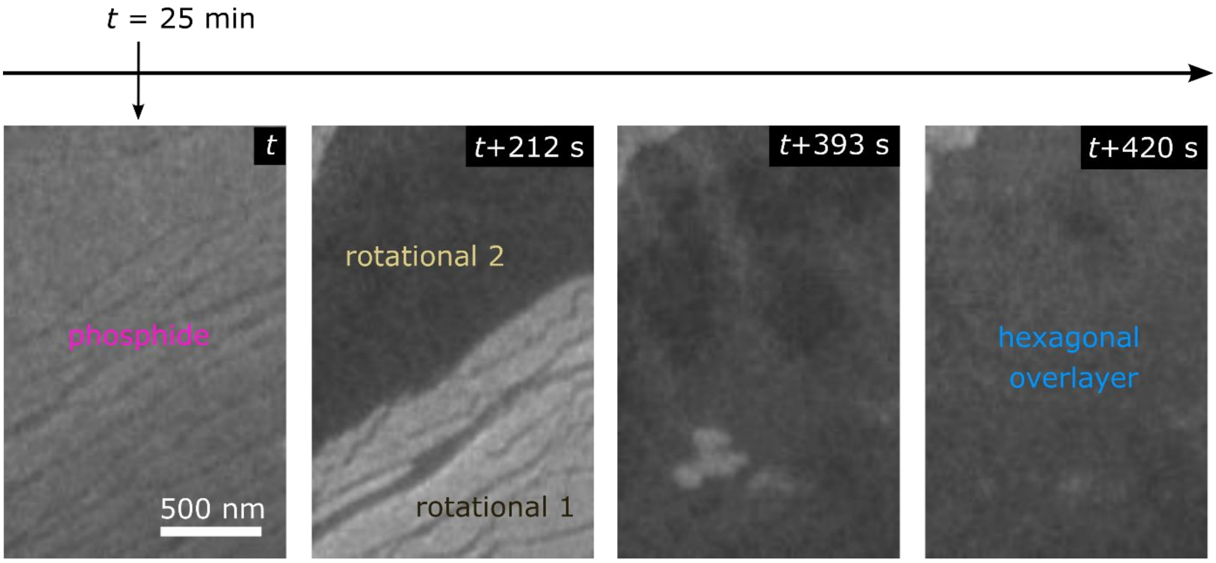

Movie S4: Energy sweep of LEED patterns from Cu(111) after the prolonged P deposition. The movie is a compilation of LEED patterns obtained while increasing *E* from 0 to 20 eV in 0.3 eV steps at *T* = 450 K after the deposition of P at ~0.038 ML/min for *t* = 240 minutes. (This results in the formation of triangular and elliptical islands on phosphorene-covered Cu(111).) The contrast in the LEED patterns is inverted to increase the visibility of the diffraction spots. The movie documents a movement of diffraction spots with changing *E*, which is indicative of a three-dimensional nature of the islands.

Movie S5: Bright-field real-time LEEM imaging of the growth of triangular (bright) and elliptical (dark) islands. In this measurement sequence, *E* is set to 7 eV to maximize the contrast between the triangular and elliptical islands and the LEEM images (width = 860 nm) are obtained at 120 frames/s (acquired at 1 frame/s) during P deposition at ~0.038 ML/min with the Cu(111) crystal held at *T* between 450-460 K.

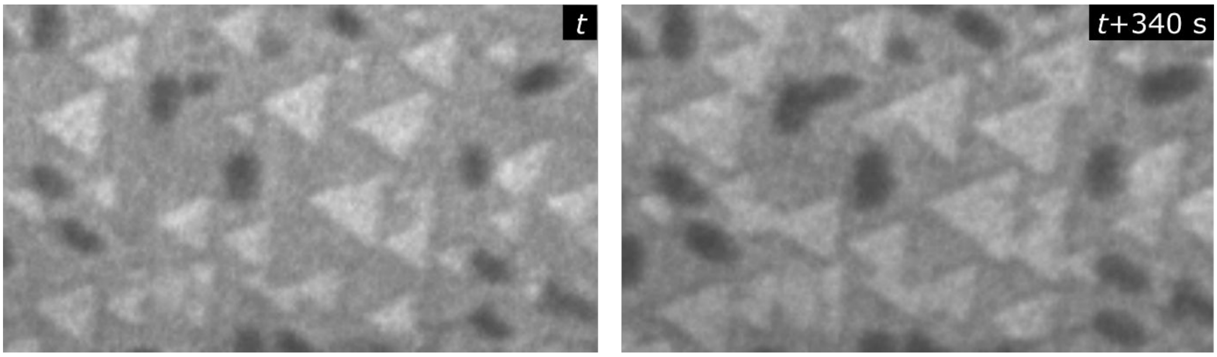

Supplement: Supplementary file 1 — nn4c11802_si_001.pdf [file nn4c11802_si_001.pdf]
